# Supplementary material for: Magnetic Resonance Imaging of Macrophage Response to Radiation Therapy
Source: Cancers (Basel). 2023 Dec 17;15(24):5874. doi: 10.3390/cancers15245874 (PMC10742077; doi:10.3390/cancers15245874)
Supplement: Supplementary file 1 [file cancers-15-05874-s001.zip › cancers-2666397-supplementary.pdf]

Magnetic Resonance Imaging of Macrophage Response to Radiation Therapy

Harrison Yang [1], Brock Howerton [2], Logan Brown [1], Tadahide Izumi [3,4], Dennis Cheek [5], J. Anthony Brandon [6], Francesc Marti [3,7], Roberto Gedaly [3,7], Reuben Adatorwovor [8] and Fanny Chapelin [2,9,*]

<u>Supplementary results</u>

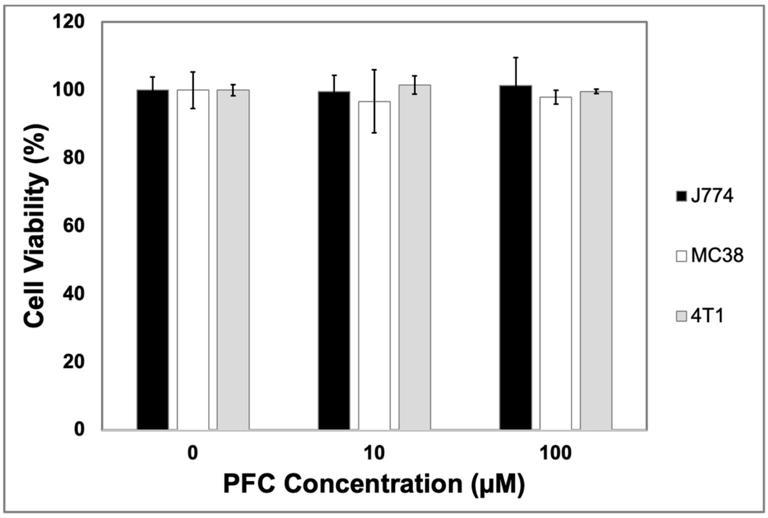

Figure S1. PFC nanoemulsion cytotoxicity assay. Results of both cancer cell lines (MC38 and 4T1) as well as mouse macrophages (J774) are displayed for different concentrations of PFC emulsion. No significant changes were seen in cell viability for all concentrations and cell lines. Data represent triplicate experiments and results are displayed as mean percentage ± standard deviation.

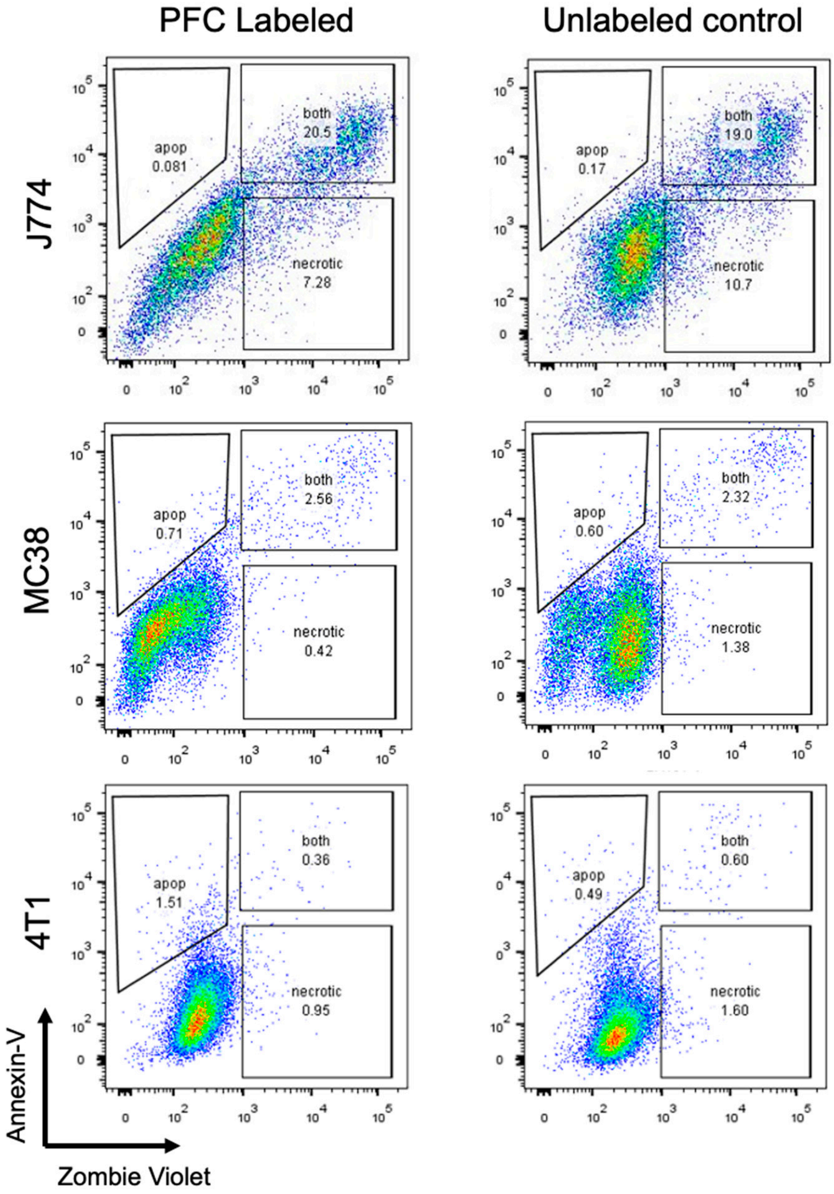

Figure S2. Flow cytometry data of cell apoptosis assays. All three cell lines were incubated for 24 hours with PFC nanoemulsion at 1mM concentration or remained unlabeled. Dual staining with Annexin-V (Y axis) and Zombie Violet (X axis) reveal no significant deleterious effect of the emulsion on any cell line, even at such concentrations.

Table S1. Quantitative results of the flow cytometry apoptosis assay. Mean and standard deviation of three samples per condition are presented. There was no notable difference in apoptotic, necrotic or both necrotic and apoptotic-positive cells in all cell lines.

		Apoptotic cells (%)	Necrotic cells (%)	Both (Apoptotic and Necrotic,%)
J774 labeled	Mean	**0.13**	**6.80**	**22.33**
	Stdev	0.05	0.89	1.85
J774 unlabeled	Mean	**0.20**	**9.46**	**19.23**
	Stdev	0.06	1.65	0.97
MC38 labeled	Mean	**0.80**	**0.53**	**3.86**
	Stdev	0.12	0.16	1.18
MC38 unlabeled	Mean	**0.50**	**1.57**	**2.58**
	Stdev	0.09	0.20	0.77
4T1 labeled	Mean	**0.71**	**1.26**	**0.29**
	Stdev	0.71	0.27	0.06
4T1 unlabeled	Mean	**0.41**	**1.32**	**0.37**
	Stdev	0.08	0.38	0.22
